# Low nutritional status links to the prevalence of pre-metabolic syndrome and its cluster in metabolically high-risk Korean adults: A cross-sectional study

**DOI:** 10.1097/MD.0000000000025905

**Published:** 2021-05-21

**Authors:** Jieun Kim, Kyoungsik Jeong, Siwoo Lee, Bok-Nam Seo, Younghwa Baek

**Affiliations:** Future Medicine Division, Korea Institute of Oriental Medicine, 1672 Yuseong-daero, Yuseong-gu, Daejeon, Republic of Korea.

**Keywords:** constitution type, diet, metabolic-syndrome, nutrition quotient

## Abstract

Diet plays a crucial role as a modifiable risk factor related to the development of metabolic syndrome (MetS) and its cluster. Constitution type of traditional Korean medicine has shown accuracy to predict the risk for MetS. We attempted to examine the association between nutritional status, pre-MetS, and its cluster in Korean adults by their constitution type.

Participants aged 30 to 55 years who had no cancer or cardiovascular diseases (CVDs) were assigned to join in the present study. Pre-MetS was defined as ≥2 of the following factors: abdominal obesity; elevated triglycerides (TG); reduced high-density lipoprotein cholesterol (HDL-C); elevated blood pressure (BP); and elevated fasting plasma glucose (FPG). Constitution type was categorized into Tae-Eumin (TE) or non-TE. Dietary assessment of the subjects were surveyed using a short-form of the food frequency questionnaire (FFQ) and the nutrition quotient (NQ), which uses 4 factors, namely, balance, diversity, moderation, and dietary behavior.

A total of 986 subjects were evaluated by constitution type with MetS status. Of these subjects, 48.6% had pre-MetS, 89.5% were obese and had the highest waist circumference (WC) in Pre-MetS TE. BP, FPG, TG were higher, while HDL-C was lower, than normal TE or non-TE both in Pre-MetS TE and non-TE. The prevalence of pre-MetS was positively associated with lower status of dietary behavior (odds ratio [ORs]: 2.153, 95% confidence interval [CI]: 1.179–3.931) while negatively related to higher vegetables and fruits intakes (ORs: 0.594, 95% CI: 0.359–0.983) in TE. Lower status of NQ had about 2 times higher risk of Pre-MetS (ORs: 1.855, 95% CI: 1.018–3.380) and abdominal obesity (ORs: 2.035, 95% CI: 1.097–3.775) in TE compared with higher status of NQ after controlling for covariates.

Poor diet was a key contributor to the development of Pre-MetS and abdominal obesity in Korean adults with TE. Customized nutrition care and integrated medicinal approaches are strongly suggested to conduct optimal preventive care for people who are vulnerable to health risk.

## 1 Introduction

Metabolic syndrome (MetS) is defined as a clustering risk factor that doubles cardiovascular disease (CVD) and fivefold increases in type 2 diabetes mellitus (T2DM).^[1]^ MetS is a pathological condition characterized by abdominal obesity, insulin resistance, elevated blood pressure, and dyslipidemia.^[2–4]^ Globally, the prevalence of MetS is estimated to be 20% to 25% of the population and has become a public health hazard.^[5]^

As a preventive approach, the constitution type of traditional Korean medicine is a diagnosis with a view of psychological, social, and physical aspects, such as bodily structure, function, and metabolism.^[6,7]^ Numerous previous studies have found that individuals of Tae-eumin (TE) type are likely to be predisposed to MetS as a metabolically high-risk group with higher waist circumference,^[8]^ elevated blood pressure,^[9]^ highest total cholesterol and triglyceride and lowest high-density lipoprotein cholesterol (HDL-C),^[10]^ insulin resistance,^[11]^ and diabetes.^[12]^ Recently, the accuracy of risk prediction for MetS^[13]^ and CVD^[14]^ has increased.

Pre-metabolic syndrome (Pre-MetS) is the earlier stage of MetS and is defined as having no less than two clusters of MetS with a high risk status preceding CVD.^[15,16]^ Lifestyle-related factors are well-known as modifiable risk factors to prevent metabolic-related diseases in people with a high risk status of MetS or CVD. Overall diet and nutritional status could be utilized to evaluate the predominant risk factors in individuals or populations that either adheres to a healthful diet or not.^[17]^ Evidence-based epidemiological studies have reported an association between dietary factors and the presence of MetS.^[18–21]^ Healthy dietary behaviors, such as a habitual plant-based soy protein diet or organic food with adherence to dietary guidelines, were negatively associated with MetS components.^[20,21]^ Meanwhile, positive link between intake of food amount, meal speed, and the prevalence of Pre-MetS and MetS has been reported.^[22]^ Recent research found that low quality of diet with western style was an independent predictor increasing MetS in metabolically unhealthy patients.^[23]^

To the best of our knowledge, no study has been designed to identify nutritional status and MetS clustering risk factors by constitution type and their associations in population-based studies. Thus, we attempted to examine the association between nutritional status, Pre-MetS, and its cluster in Korean adults by constitution type.

## 2 Material and methods

### 2.1 Study design and population

The Korean Medicine Daejeon Citizen Cohort (KDCC) study^[24]^ is the first prospective ongoing cohort study to assess associations with lifestyle factors and chronic diseases according to Korean medicine types. All study procedures were conducted by well-trained expert interviewers and researchers from the Korea Institute of Oriental Medicine (KIOM), Gallup Korea and Korean Medicine Hospital of Daejeon University. Previously, we published a study protocol^[24]^ and explained the sample size calculations to detect chronic diseases and statistical power of analysis. All data collected from the cohort study were handled by using the secure, web-based Korean Medicine Data Center (KDC) electronic data capture system by the KDC of the KIOM. We only analyzed the cross-sectional survey data after obtaining the permission of the KDC administration.

**Figure 1:**
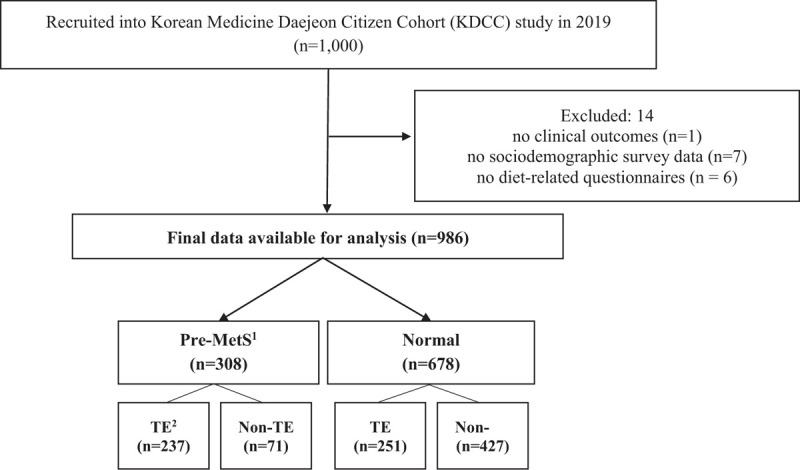
Flow diagram illustrating the selection of subjects for analysis. 1. Pre-Metabolic syndrome (MetS) was defined as having two or more of the following: elevated triglycerides: ≥150 mg/dL or drug treatment for elevated triglycerides as an alternate indicator. Reduced high-density lipoprotein cholesterol (HDL-C): <40 mg/dL in men and <50 mg/dL in women or drug treatment for reduced HDL-C as an alternate indicator. Elevated fasting glucose: ≥100 mg/dL or drug treatment of elevated glucose as an alternate indicator. Elevated blood pressure: systolic BP (SBP) ≥130 mm Hg or diastolic BP (DBP) ≥85 mm Hg or on antihypertensive medication. Elevated waist circumference: ≥90 cm in men and ≥85 cm in women Cut-off point for waist circumference was based on the Asia-Pacific. World Health Organization guideline. 2. Constitution type was categorized into 2 groups; Non-TE = So-eumin and So-yangin, TE = Tae-eumin.

The population included men and women aged ≥30 years and ≤55 years who were residents of Daejeon at enrollment in the KDCC between June 2017 and December 2019. Participants who had problems reading and understanding literacy or following study instructions and were diagnosed with cancer(s) or CVDs, such as myocardial infarction, angina, or stroke, were excluded. A total of 2000 participants were enrolled in the study at baseline. We began nutritional assessment in 2019; therefore, only 1000 patients were assessed for their nutritional status, and those of 8 were missing their data in clinical outcomes (n = 1), sociodemographic survey data (n = 7), and diet-related questionnaires (n = 6). Finally, 986 participants were included in the present study. Tae-yangin was not included in the analysis because of the low rates (<0.1%) of all participants. Participants were categorized into 2 groups: TE/non-TE (So-yangin [SY] and So-eumin [SE]) with Pre-MetS or TE/non-TE with normal groups (Fig. 1).

Informed consent was obtained from all participants when data were collected. The Institutional Review Board at the KIOM and Dunsan Korean Medicine Hospital of Daejeon University reviewed and approved this study (IRB No. I-1703/002–002, DJDSKH-17-BM-12).

### 2.2 Definition of MetS cluster

To determine the metabolic status of the participants, the following diagnosis and guidelines were followed. Pre-MetS is defined by the National Cholesterol Education Program-Adult Treatment Panel III (NCEP-ATP III)^[2]^ if participants have ≥2 of the following factors:

Abdominal obesity based on waist circumference (WC) with cutoff points specific to South Koreans (WC ≥90 cm in men and ≥85 cm in women) plus any of the following: elevated triglycerides (TG) ≥150 mg/dL or specific treatment for this lipid abnormality; reduced <40 mg/dL in men and <50 mg/dL in women or drug treatment for this lipid abnormality; elevated blood pressure (BP), systolic BP (SBP) ≥130 or diastolic BP (DBP) ≥85 mmHg or treatment of previously diagnosed hypertension; and elevated fasting plasma glucose (FPG) ≥100 mg/dL, or previously diagnosed type 2 diabetes.

### 2.3 Sasang constitutional (SC) type

The Korean Sasang constitutional diagnostic questionnaire (KS-15)^[25]^ was employed to assess the individual constitution of the participants. The KS-15 was developed by the KIOM based on information on body shape, temperament, and symptoms.^[25]^ The KS-15 questionnaire consists of 15 items of anthropometric awareness of height and weight, 6 questions of personality (broad-minded/delicate, act quick/slow, active/passive, extraverted/introverted, masculine/feminine, excitable/rational), and 8 symptom-related questions of physiological functions (digest well, appetite, much sweat, feeling after seating, abdominal tension during a bowel movement, urination per night during sleep time, dislike cold and heat, preference for temperature when drinking water) (Cronbach α = 0.630). The KS-15 is a well-validated short form of assessment tool for constitution type that adapts body mass index (BMI) and age- and sex-specific weighted values for higher coincidence with clinical relevance.^[25]^ We defined the 2 constitution types of TE type or non-TE (SY and SE) to compare the differences in the metabolic outcomes and nutritional status among the 3 main types of constitution.

### 2.4 Sociodemographic characteristics, anthropometric measurements, and MetS cluster

Sociodemographic characteristics for the study population, including age, sex, education level, marital status, household income, and disease history, were surveyed at baseline.

Anthropometric measurements to the nearest 0.1 kg or 0.1 cm (i.e., height, weight, WC, SBP, and DBP) were taken by trained personnel with the participants dressed lightly without their shoes before height and weight measurement with the measuring station BSM370 and Inbody 770 (Biospace, Korea). Body mass index (BMI) was calculated as weight in kg divided by height in meters squared (m^2^). BP was measured using an automatic blood pressure cuff (FT-500R PLUS, Jawon Medica, Korea) after 5 to 10 minutes of rest, and the mean value of the 2 measurements was used for analysis. WC was measured with a tape measure (Hoechst mass-Rollfix, Germany) according to World Health Organization (WHO) guidelines.

Laboratory data (i.e., TG, HDL-C, and FPG) were taken after overnight fasting. After 30 minutes of incubation, collected blood samples were centrifuged for 10 minutes at 3450 rpm, and all samples were transported to the Seoul Clinical Laboratories (Seoul, Korea) within 24 hours.

### 2.5 Short-form of food frequency questionnaire

Dietary intake using a semi-quantitative food frequency questionnaire (FFQ), which was validated in previous study. Subjects were asked to report their frequency (9 categories ranging from “never or rare” to “three times per day”) and portion size (3 or 4 specified portion sizes) of each food item over the past year.

The short form of FFQ consisted of groups of grains (rice, mixed grains, noodles, bread, potatoes, and sweet potatoes), proteins (beef, pork, fish, beans, tofu, and eggs), dairy, vegetables, and fruits, seaweeds, coffee and tea, alcohol, and fast foods. Reported frequency of each food item, converted to daily gram intake of macronutrients, as well as total energy, using the Computer Aided Nutritional Analysis Program (CAN Pro, Version 5.0, The Korean Nutrition Society, 2015).

### 2.6 Nutrition quotient

The nutritional status of the participants was assessed using the nutrition quotient (NQ),^[26]^ which was developed by the Korea Nutrition and Health Society. The NQ was designed to evaluate diet quality, eating attitudes, and behaviors in adults through 21 items which have 4 factors: balance (intake frequency of fruits, eggs, bean/bean products, dairy, nuts, seafood, and breakfast), diversity (number of vegetable dishes [excluding kimchi] at each meal, frequency of drinking water, and refusal of specific food items) moderation (intake frequency of fast food, ramyeon, night snack, eating out or delivered food, sweets and bakery, processed beverage), and dietary behavior (check nutrition label, efforts to healthy eating habits, perception level of one's health, exercise over 30 minutes, washing hands before eating), and its total score. The scores of all 4 factors were summed to yield the total NQ score, ranging from 0 to 100. Three different grades (high: 75–100% tile, middle: 25% to <75% tile, low: 0% to <25% tile) were assigned according to the total NQ score, and a higher score indicated better dietary behaviors.

### 2.7 Statistical analysis

Descriptive analyses of the participants according to their constitution type and MetS status are presented in Tables 1 and 2. Group differences were evaluated using chi-square tests for categorical variables; age, sex, marital status, education, household income, weight status, disease history, Pre-MetS, and NQ status and multiple comparison of one-way analysis of variance and analysis of covariance (ANCOVA) for means of age and clinical outcomes in Table 1 and energy, macronutrients FFQ, and total NQ and its factors in Table 2.

**Table 1:** Group differences in sociodemographic characteristics and MetS cluster of the subjects according to constitution type^∗^ with MetS status^†^.

	All (n = 986)		Pre-MetS (n = 308)		Normal (n = 678)		
Characteristics	TE (n = 488)	Non-TE (n = 498)	TE (n = 237)	Non-TE (n = 71)	TE (n = 251)	Non-TE (n = 427)	*P*
Age, y	44.1 ± 0.3	44.6 ± 0.3	45.6 ± 0.4^b^	48.1 ± 0.8^¶^	42.6 ± 0.4^c^	44.0 ± 0.3^c^	<.0001
30–44 years (n, %)	252 (51.6)	251 (50.4)	98 (41.4)	22 (31.0)	154 (61.4)	229 (53.6)	<.0001
45–55 years	236 (48.4)	247 (49.6)	139 (58.6)	49 (69.0)	97 (38.6)	200 (46.4)	
Gender (%)							
Male	162 (33.4)	97 (19.5)	95 (40.1)	29 (40.9)	67 (26.7)	68 (15.9)	<.0001
Female	326 (66.6)	401 (80.5)	142 (59.9)	42 (59.2)	184 (73.3)	359 (84.1)	
Marital status (%)							
Unmarried	47 (9.6)	38 (7.6)	17 (7.2)	2 (2.8)	30 (12.0)	36 (8.4)	.206
Married	415 (85.0)	437 (87.8)	208 (87.8)	67 (94.4)	207 (82.5)	370 (86.7)	
Never-married	26 (5.3)	23 (4.6)	12 (5.0)	2 (2.8)	14 (5.5)	21 (4.9)	
Education (%)							
High school lower levels	173 (35.5)	169 (33.9)	101 (42.6)	20 (28.2)	72 (28.7)	149 (34.9)	.008
College and higher levels	315 (64.5)	329 (66.1)	136 (57.4)	51 (71.8)	179 (71.3)	278 (65.1)	
Household income^‡^ (%)							
Low	121 (25.0)	92 (18.7)	66 (28.0)	6 (8.57)	55 (22.1)	86 (20.4)	.004
Middle	326 (67.2)	344 (69.9)	148 (62.7)	53 (74.6)	178 (71.5)	291 (69.1)	
High	38 (7.8)	56 (11.4)	22 (9.3)	12 (16.9)	16 (6.4)	44 (10.5)	
Weight status^§^ (%)							
BMI, kg/m^2^ <25	143 (29.3)	480 (96.4)	25 (10.5)	62 (87.3)	118 (47.0)	418 (97.9)	<.0001
25 ≤ BMI, kg/m^2^	345 (70.7)	18 (3.6)	212 (89.5)	9 (12.7)	133 (53.0)	9 (2.1)	
Disease history (%)							
Hypertension	123 (25.2)	58 (11.7)	106 (44.7)	32 (45.1)	17 (6.8)	26 (6.1)	<.0001
Diabetes	20 (4.1)	6 (1.2)	20 (8.4)	5 (7.0)	0 (0.0)	1 (0.2)	<.0001
Dyslipidemia	144 (29.5)	96 (19.3)	114 (48.1)	39 (54.9)	30 (12.0)	8 (13.4)	<.0001
MetS cluster (mean ± SE)							
WC, cm	87.6 ± 8.5	75.9 ± 6.2	92.4 ± 0.4^¶^	80.5 ± 0.8^c^	84.7 ± 0.4^b^	76.9 ± 0.3^d^	<.0001
SBP, mmHg	122.8 ± 15.1	115.9 ± 14.0	130.1 ± 0.8^¶^	130.4 ± 1.5^¶^	117.5 ± 0.8^b^	114.9 ± 0.7^b^	<.0001
DBP, mmHg	76.4 ± 12.6	70.9 ± 11.0	82.2 ± 0.7^¶^	82.0 ± 1.3^¶^	73.1 ± 0.7^b^	71.4 ± 0.6^b^	<.0001
Fasting plasma glucose, mg/dL	86.6 ± 17.8	81.7 ± 13.1	91.3 ± 1.0^¶^	91.6 ± 1.8^¶^	83.3 ± 1.0^b^	81.1 ± 0.8^b^	<.0001
HDL-C, mg/dL	53.5 ± 12.6	61.3 ± 14.2	47.3 ± 0.8^c^	47.7 ± 1.4^c^	57.5 ± 0.8^b^	61.6 ± 0.7^¶^	<.0001
TG, mg/dL	142.3 ± 113.9	108.6 ± 80.9	195.8 ± 5.7^¶^	202.9 ± 10.4^¶^	108.0 ± 5.7^b^	109.1 ± 4.7^b^	<.0001

**Table 2:** Group differences in nutritional status of the subjects according to constitution type with Mets status.

	Pre-MetS (n = 308)		Normal (n = 678)		
Variables	TE (n = 237)	Non-TE (n = 71)	TE (n = 251)	Non-TE (n = 427)	*P*
Energy, kcal/d^∗^					
Men	2222.7 ± 69.5	2417.0 ± 128.6	2166.3 ± 84.7	2277.0 ± 81.7	.419
Women	2115.8 ± 58.0	2034.9 ± 106.8	2004.3 ± 50.8	1952.5 ± 36.3	.125
Macronutrients^†^					
Carbohydrates, g	324.5 ± 7.2	330.3 ± 13.2	311.1 ± 7.3	313.9 ± 6.0	.403
Fat, g	54.1 ± 1.6	53.6 ± 2.8	52.8 ± 1.6	50.3 ± 1.3	.231
Protein, g	72.7 ± 1.7	72.8 ± 3.2	70.4 ± 1.8	68.3 ± 1.5	.215
C : F : P, %	59.7:22.1:13.2	59.4:21.6:13.1	59.5:22.6:13.4	60.4:21.8:13.1	N/S
FFQ, times/wk^†^					
White rice	16.1 ± 0.4	16.7 ± 0.7	15.8 ± 0.4	16.5 ± 0.3	.358
Mixed rice	7.4 ± 0.5	7.2 ± 0.9	6.9 ± 0.5	7.5 ± 0.4	.752
Noodles/bread	3.6 ± 0.2	4.0 ± 0.4	3.2 ± 0.2	3.4 ± 0.2	.364
Potatoes/sweet potatoes	1.7 ± 0.2	1.8 ± 0.3	1.5 ± 0.2	1.6 ± 0.2	.870
Beans/tofu	6.0 ± 0.4	5.1 ± 0.8	5.8 ± 0.4	5.0 ± 0.3	.177
Fish	1.9 ± 0.1	2.0 ± 0.3	1.9 ± 0.1	1.8 ± 0.1	.839
Beef/pork	2.0 ± 0.1	2.1 ± 0.3	2.0 ± 0.2	1.8 ± 0.1	.677
Poultry	1.6 ± 0.1	1.8 ± 0.2	1.5 ± 0.1	1.5 ± 0.1	.639
Eggs	4.2 ± 0.2^§^^,b^	3.8 ± 0.4^§^^,b^	4.5 ± 0.2^§^	3.8 ± 0.2^b^	.043
Vegetables/fruits	10.0 ± 0.6	12.0 ± 1.2	11.0 ± 0.6	10.5 ± 0.5	.423
Seaweeds	3.5 ± 0.3	3.5 ± 0.5	3.1 ± 0.3	3.2 ± 0.2	.673
Milk/yogurt	4.7 ± 0.4	4.3 ± 0.7	6.2 ± 0.5	5.8 ± 0.4	.249
Total NQ^†^^,^^‡^	52.8 ± 0.6	53.9 ± 1.0	54.8 ± 0.5	53.2 ± 0.4	.047
Balance	29.4 ± 0.9^§^^,b^	31.5 ± 1.6^§^^,b^	32.6 ± 0.9^§^	29.6 ± 0.7^b^	.021
Diversity	58.8 ± 1.0^§^^,b^	58.4 ± 1.8^§^^,b^	59.6 ± 1.0^§^	55.5 ± 0.7^b^	.004
Moderation	73.2 ± 0.8	74.7 ± 1.5	73.4 ± 0.8	74.4 ± 0.6	.573
Dietary behavior	44.0 ± 0.9^b^	45.3 ± 1.6^§^^,b^	48.6 ± 0.9^§^	48.0 ± 0.7^§^	.001

**Figure 2:**
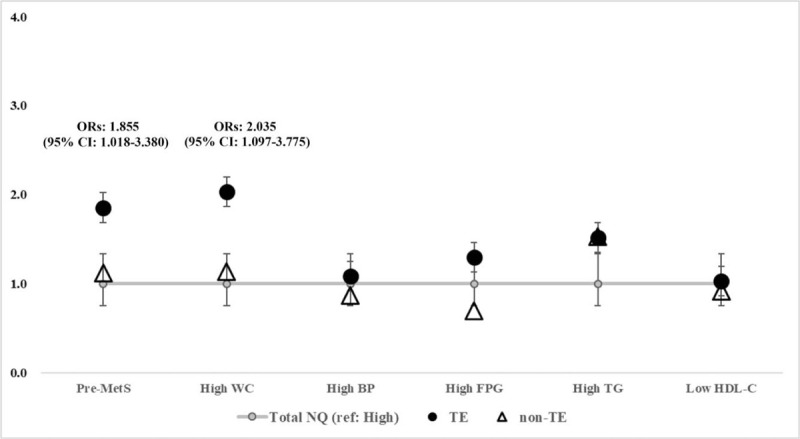
Associations between low status of the total NQ^1^ and prevalence of Pre-MetS and its cluster by constitution type^2^ of the subjects. ^1^Total Nutrient quotient (NQ): Three different grades (high: 75–100% tile, middle: 25% to <75% tile, low: 0% to <25% tile) were assigned according to the total NQ score, and a higher score indicated better dietary behaviors. ^2^Constitution type was categorized into 2 groups; TE: Tae-eumin (TE); Non-TE: So-eumin (SE) and So-yangin (SY) ^∗^*P* for trend was tested across 3 levels (low–high) of the 4 factors of NQ by including the median score as a continuous measure in the regression model. Adjusted covariates were included age, sex, education level, household income, and weight status.

We fit a multivariate logistic regression model to examine the association between dietary behavior, vegetables and fruits intakes, and Pre-MetS prevalence in Table 3, nutritional status and Pre-MetS prevalence and its cluster in Fig. 2 by constitution type. All models included age, sex, education level, household income, weight status (Model II a) and energy intake (Model II b); however, we did not control for health-related variables, such as smoking, alcohol consumption, and physical activity, because there are studies^[27,28]^ suggesting that they are in the causal pathways between nutritional status and MetS.

**Table 3:** Dietary factors associated with Pre-MetS according to constitution type of the subjects.

	Model I ORs (95% CI)		Model II ORs (95% CI)	
	TE	Non-TE	TE	Non-TE
Dietary behavior	(ref: High)^∗^			
Middle	1.382 (0.857–2.229)	1.228 (0.628–2.402)	1.383 (0.825–2.316)	1.185 (0.596–2.357)
Low	**2.283 (1.306–3.991)**	2.070 (0.964–4.446)	**2.153 (1.179–3.931)**	**2.490 (1.132–5.476)**
*P* for trend	**0.004**	0.0642	**0.012**	**0.027**
Vegetables and fruits	(ref: Low)^†^			
Middle	0.787 (0.502–1.236)	0.640 (0.316–1.295)	0.628 (0.369–1.071)	0.501 (0.231–1.087)
High	**0.608 (0.386–0.958)**	0.999 (0.524–1.903)	**0.594 (0.359–0.983)**	0.852 (0.435–1.669)
*P* for trend	0.421	0.172	0.118	0.250

We performed all analyses using SAS 9.4 (SAS Institute, Cary, NC); all analyses were 2-tailed, and a *P*-value of .05 was considered to be significant.

## 3 Results

### 3.1 Group differences in sociodemographic characteristics and MetS cluster of the subjects according to constitution type with Mets status

In total, 986 subjects were evaluated according to their constitution types with MetS status. Approximately one-third of participants had Pre-MetS, and 48.6% had TE. Significant group differences were observed between TE and non-TE with Pre-MetS (*P* < .0001). All sociodemographic variables, such as age, sex, education level, and household income, were significantly different between the 4 groups except marital status. Both TE and non-TE with Pre-MetS were older (*P* < .0001) and more were men (40.1% of Pre-MetS TE and non-TE vs 26.7% of normal TE, 15.9% of non-TE, *P* < .0001) than normal TE and non-TE groups. Pre-MetS TE was shown to be lower in education (*P* = .008) and household income (*P* = .004) than in their counterparts (Table 1).

With regard to obesity (89.5% were obese) and MetS-related disease history (44.7% for hypertension, 8.4% for diabetes, and 48.1% had dyslipidemia), significantly higher rates were observed in Pre-MetS TE.

Regarding the MetS cluster, WC (Pre-MetS TE: 92.4 ± 0.4^a^, *P* < .0001) was the highest in Pre-MetS TE. Both Pre-MetS TE and non-TE were high in SBP (mmHg), fasting plasma glucose (mg/dL), and TG (mg/dL) but lower in HDL-C (mg/dL) (*P* < .0001) (Table 1).

### 3.2 Group differences in nutritional status of the subjects according to constitution type with MetS status

Dietary intakes and nutritional status of the participants is presented in Table 2. No significant differences in energy (kcal/d), macronutrients (g/d), and foods intake (times/wk) except eggs between the 4 groups. Total NQ scored highest in the normal TE group compared with the other groups (*P* = .047) after adjusting for age and sex. Balance factors, which include intakes of fruit, dairy, beans, eggs, seafood, nuts and breakfast, and diversity factors were higher in the normal TE than in their counterparts (*P* < .05). Meanwhile, low status of the moderation (*P* < .01) and lower scoring in dietary behavior were seen in the Pre-MetS TE (*P* < .001) (data not shown) (Table 2).

### 3.3 Nutritional status associated with Pre-MetS according to constitution type of the subjects

The association between dietary behavior, vegetables and fruits intakes, and Pre-MetS prevalence is presented in Table 3. Lower status of dietary behavior was positively associated with the prevalence of pre-MetS (compared with high status of dietary behavior) in TE (ORs: 2.153, 95% CI: 1.179–3.931) and non-TE (ORs: 2.490, 95% CI: 1.132–5.476). On the contrary, higher vegetables and fruits intakes were negatively associated with the prevalence of pre-MetS in TE (ORs: 0.594, 95% CI: 0.359–0.983) (Table 3).

Covariate-adjusted multiple logistic regression analysis was performed to assess the association between total NQ and MetS cluster in Fig. 2. About 2 folds of the prevalence of Pre-MetS (ORs: 1.855, 95% CI: 1.018–3.380) and abdominal obesity (ORs: 2.035, 95% CI: 1.097–3.775) were seen in the low status of total NQ compared with that of high status in TE (Fig. 2).

## 4 Discussion

This study examined the association between nutritional status and Pre-MetS and its cluster by constitution type in South Korean adults. With regard to the nutritional status of the groups, a higher status of total NQ, balance, and diversity were observed in the normal TE group than in the other groups. Conversely, lower status of moderation and dietary behavior showed in Pre-MetS TE. The prevalence of pre-MetS was positively associated with lower status of dietary behavior (ORs: 2.153, 95% CI: 1.179–3.931) while negatively related to higher vegetables and fruits intakes (ORs: 0.594, 95% CI: 0.359–0.983) in TE. Lower status of NQ had about 2 times higher risk of Pre-MetS (ORs: 1.855, 95% CI: 1.018–3.380) and abdominal obesity (ORs: 2.035, 95% CI: 1.097–3.775) in TE compared with higher status of NQ after controlling for covariates.

We found a high Pre-MetS prevalence in participants, particularly 48.6% of TEs. Participants who had Pre-MetS were older and were more likely to be men in both TE and non-TE groups. Of 89.5% of Pre-MetS patients, TE was obese, and those who had the highest WC had lower levels of education and income than did participants in the other groups. SBP, FPG, and TG were higher, while HDL-C was lower, than normal TE or non-TE both in Pre-MetS TE and non-TE.

According to SC theory, the TE type is prone to obesity with lung-liver seesaws (weak state of the lung system and strong liver system), which cause an energy imbalance state physiologically through anabolic and catabolic pathways.^[29]^ For instance, fat mass and obesity-associated genetic effects were explored to find sensitivity to energy intake due to energy preservation by constitution type.^[30]^ Previous studies reported harmful predisposing genetic factors^[31]^ in TE and a high risk of lipid ratio (LDL-C:HDL-C ratio) compared with those of the non-TE group.^[32]^ In line with that result, present study showed the possibilities of constitution type as a preeminent diagnostic methodology in individual participants who were at high risk by reducing the risk of MetS or CVD.

Data from cohort studies and clinical settings reported a high prevalence of MetS by SC type (14.1% for the SE type, 26.7% for the SY type, and 50.8% for the TE type).^[33]^ A similar prevalence was reported in a previous large population-based study in TE (pre-MetS: 48.2%, MetS: 41.2%).^[22]^ Another study reported highly elevated FBG, SBP, DBP, TG, and WC and depressed HDL-C in the TE group compared with the non-TE group (SY and SE groups) from 1617 outpatients.^[6]^ Dyslipidemic indices, such as high TC and LDL-C and low HDL-C, were detected in the TE group.^[10]^ In agreement with previous reports, almost half of TE patients had Pre-MetS with abdominal obesity and a higher degree of body mass index. However, a higher risk of MetS cluster—higher in SBP, FPG, TG and lower in HDL-C was seen in both Pre-MetS TE and Non-TE. These findings further suggest the notable status of Pre-MetS in both TE and non-TE patients in the present study. Most studies have shown that 3 constitution types directly describe ORs^[10,12,33]^ or RR^[34]^ for MetS-related disease by comparing TE and SY or SE groups. Because focusing on constitution type only might lose physiological insights into MetS in individuals, we screened individual constitution types with MetS status, not only the high-risk constitution type (normally TE) but also in Pre-MetS non-TE patients who had latent risk factors.

In a previous multiethnic cohort study, fast eaters consumed more calories (105 kcal/d more, *P* = .034) and had associations with general (multivariable OR: 2.2; 95% CI, 1.8–2.6; *P* < .001) and abdominal (OR: 1.8; 95% CI, 1.5–2.2; *P* < .001) obesity than slower eaters.^[19]^ Consistently, greater amount of food intake were 1.95 times higher in the Pre-MetS group (95% CI, 1.13–3.36) and 2.48 times higher in the MetS group (95% CI, 1.44–4.27) than that of normal in TE group.^[22]^ Interestingly, we found the highest nutritional status, particularly a balanced and diverse diet with fruit, vegetables, dairy, beans, eggs, seafood, nuts and breakfast consumption, in normal TE. In contrast, 37% of Pre-MetS TEs were in the low status of moderation in ramyeon, confectionery, SSBs, fast food, or eating out and night-eating. Furthermore, healthful dietary behaviors, such as check a food labeling, washing hands before a meal, and self-consciousness regarding health, was the lowest in Pre-MetS TE (data not shown). This finding highlighted that even people who had the same constitution type could change their physiological or psychological weakness or strength by adhering to healthful dietary habits and lifestyle management. Therefore, continuous nutritional management in healthy diet and nutritional education, not only focusing on single food items, is strongly required to prevent chronic diseases and life-long health to target vulnerable individuals.

Population-based prospective cohort studies presented that the association of habitual consumption of soy protein (the lowest to the highest quintile of average soy protein intake; incidence rate ratios = 0.73, 95% CI = 0.53–1.01) and reduced risk of MetS^[20]^ and the highest quintile organic food with adherence to dietary guidelines had a 35% lower risk of T2D (95% CI = 0.43–0.97).^[21]^ An evidence-based meta-analysis^[35]^ showed that both low-carbohydrate and low-fat diets were associated with clinically meaningful weight reduction when compared with no diet over a 12-month period. Furthermore, greater weight loss (12 months: 1.08 kg [95% CI, 1.82–3.96] to 6 months: 3.23 kg [95% CI, 2.23–4.23]) was shown in the behavioral support in the meta-regression model. Our results showed that a positive association between lower nutritional status including dietary behavior while negative association with higher vegetable and fruits intakes with the prevalence of Pre-MetS in TE. Particularly, lower status of dietary behavior was positively related to the Pre-MetS risk in non-TE. These results reflect the important role of dietary behavior as a key modulator in adherence to dietary guidelines and dietary reference intake (DRI, The Korean Nutrition Society, 2020) with healthful foods (such as vitamins and mineral-rich fruit and vegetables, low-fat or plant-based proteins, and whole grains) by considering constitution types, individual physical activities level and clinical status. Lastly, we observed that low status of total NQ was positively associated with the prevalence of abdominal obesity in TE. Abdominal obesity is regarded as a fundamental clinical risk factor which reflects higher visceral fat accumulation in the abdomen area contributes to insulin resistance, diabetes, and CVD. For applying clinical nutrition management^[36–40]^ such as individualized calorie restriction for weight control; decrease total calories from alcohol and carbohydrates for glycemic control; reduce intake of sodium, simple sugars, saturated fat, and total fat for lowering BP; increase consumption of fruit and vegetables by decreasing MetS and CVDs onset in metabolically high-risk population. Therefore, it is recommended that population-based positive behavioral support and self-efficacy accomplish a healthy nutritional status by considering, genetic and environmental characteristics.

This study has a number of limitations. This report describes a cross-sectional analysis from a prospective cohort study that includes only baseline data; therefore, the causal relationship between nutritional status and MetS cluster by constitution type could not be evaluated. Second, food intakes was obtained from short form of FFQ, to consider the continuous participation rate and convenience of the participants thereby participants’ usual food intake might not be fully reflected in the present data. Further study will be needed to consider real food consumption in the target population.

Our study also has number of strengths. To the best of our knowledge, the present study is the first to explore the relationship of nutritional status, pre-MetS prevalence and its cluster in Korean adults by constitution type. We also proposed an integrated view of Korean-Western medicine as an optimal prevention medicine approach to link tailored nutritional treatment. It is worth noting an evidence-based approach to provide nutritional support to consider individual strength and weakness in their diet and health.

## 5 Conclusion

Low nutritional status was a key contributor to the development of Pre-MetS and abdominal obesity in Korean adults with TE. Our findings suggest tailored nutritional management for balanced and vegetable/fruit rich diverse diets that could reduce the predisposed risk factors related to the development of cardiovascular and metabolic diseases in TE. To address the global and public health burden, customized and integrated medicinal approaches are strongly suggested to provide optimal preventive care for people who are vulnerable to health risk.

## Author contributions

**Conceptualization:** Jieun Kim.

**Data curation:** Kyoungsik Jeong, Bok-Nam Seo.

**Formal analysis:** Jieun Kim.

**Funding acquisition:** Siwoo Lee.

**Investigation:** Bok-Nam Seo.

**Methodology:** Jieun Kim.

**Project administration:** Siwoo Lee, Younghwa Baek.

**Resources:** Kyoungsik Jeong.

**Software:** Kyoungsik Jeong.

**Supervision:** Younghwa Baek.

**Writing – original draft:** Jieun Kim.

**Writing – review & editing:** Siwoo Lee, Younghwa Baek.
